# Repeated transspinal stimulation decreases soleus H-reflex excitability and restores spinal inhibition in human spinal cord injury

**DOI:** 10.1371/journal.pone.0223135

**Published:** 2019-09-26

**Authors:** Maria Knikou, Lynda M. Murray

**Affiliations:** 1 Klab4Recovery Research Laboratory, Department of Physical Therapy, College of Staten Island, The City University of New York, Staten Island, New York, United States of America; 2 PhD Program in Biology and Collaborative Neuroscience Program, Graduate Center of The City University of New York, New York, New York, United States of America; Szegedi Tudomanyegyetem, HUNGARY

## Abstract

Transcutaneous spinal cord or transspinal stimulation over the thoracolumbar enlargement, the spinal location of motoneurons innervating leg muscles, modulates neural circuits engaged in the control of movement. The extent to which daily sessions (e.g. repeated) of transspinal stimulation affects soleus H-reflex excitability in individuals with chronic spinal cord injury (SCI) remains largely unknown. In this study, we established the effects of repeated cathodal transspinal stimulation on soleus H-reflex excitability and spinal inhibition in individuals with and without chronic SCI. Ten SCI and 10 healthy control subjects received monophasic transspinal stimuli of 1-ms duration at 0.2 Hz at subthreshold and suprathreshold intensities of the right soleus transspinal evoked potential (TEP). SCI subjects received an average of 16 stimulation sessions, while healthy control subjects received an average of 10 stimulation sessions. Before and one or two days post intervention, we used the soleus H reflex to assess changes in motoneuron recruitment, homosynaptic depression following single tibial nerve stimuli delivered at 0.1, 0.125, 0.2, 0.33 and 1.0 Hz, and postactivation depression following paired tibial nerve stimuli at the interstimulus intervals of 60, 100, 300, and 500 ms. Soleus H-reflex excitability was decreased in both legs in motor incomplete and complete SCI but not in healthy control subjects. Soleus H-reflex homosynaptic and postactivation depression was present in motor incomplete and complete SCI but was of lesser strength to that observed in healthy control subjects. Repeated transspinal stimulation increased homosynaptic depression in all SCI subjects and remained unaltered in healthy controls. Postactivation depression remained unaltered in all subject groups. Lastly, transspinal stimulation decreased the severity of spasms and ankle clonus. The results indicate decreased reflex hyperexcitability and recovery of spinal inhibitory control in the injured human spinal cord with repeated transspinal stimulation. Transspinal stimulation is a noninvasive neuromodulation method for restoring spinally-mediated afferent reflex actions after SCI in humans.

## Introduction

Electrical stimulation is commonly used to promote recovery of motor function in upper motoneuron lesions. Stimulation of the primary motor cortex, spinal cord, peripheral nerve(s), and muscle(s) has been employed to improve sensorimotor function after spinal cord injury (SCI) [1–6]. Transcutaneous spinal cord (termed here transspinal) stimulation delivered via single monophasic pulses generates transspinal evoked potentials (TEPs) simultaneously in both legs of individuals with and without SCI that have distinct characteristics regarding their latency, duration, shape, and spinal integration [7–10]. We have recently demonstrated that TEPs summate in surface electromyogram (EMG) with spinal (H reflex) and peripheral (M wave) responses [11], supporting for a specific control of transspinal stimulation on spinal proprioceptive neuronal pathways. Specifically, the soleus TEP summates with the soleus H reflex when these two action potentials interact at the peripheral nerve axons [11], while at long stimulation delays the H reflex is depressed or completely abolished [7,11]. A single session of transspinal stimulation decreased ankle clonus and spasticity in a few cases of incomplete or complete SCI [12–14], while multiple sessions improved responsiveness of motoneurons spanning multiple spinal segments after SCI in humans [10].

Electrophysiological and mathematical modeling studies have provided convincing evidence that stimulation alters the excitability state of spinal motoneurons by altering their properties. Extracellularly electrical fields, produced by alternated current, reduces the motoneuron firing rate by decreasing the magnitude of dendritic persistent inward currents [15,16]. More importantly, computer modeling of neurons with SCI characteristics showed that stimulation inactivates the sodium channels in the soma and initial segment, blocks the action potentials from propagating through the axon, and reduces the motoneuron firing rate through stimulation-induced hyperpolarization [15,16]. Taken altogether, transspinal stimulation may be an effective therapeutic strategy to regulate motoneuron excitability after SCI in humans.

Collectively, the objectives of this study were to establish the effects of transspinal stimulation on soleus H-reflex excitability and spinal inhibition. We hypothesized that transspinal stimulation at intensities that produces motoneuron depolarization over multiple segments can decrease H-reflex excitability by directly affecting the strength of monosynaptic depolarization of motoneurons. We further hypothesized that H-reflex excitability and spinal inhibition will improve more in individuals with motor incomplete SCI compared to motor complete SCI. To test our hypotheses, the soleus H-reflex recruitment curves, soleus H-reflex depression at varying stimulation frequencies, termed here as homosynaptic depression, and soleus H-reflex depression in response to tibial nerve paired stimuli, termed here as postactivation depression, were assessed before and one or two days after repeated transspinal stimulation in healthy control subjects and individuals with chronic motor incomplete and complete SCI.

## Materials and methods

### Participants

All experimental and stimulation procedures were performed according to the Declaration of Helsinki after full Institutional Review Board (IRB) approval by the City University of New York IRB-wide Biomedical Committee (IRB Number 515055). Ten individuals with chronic SCI (Table 1) and 10 healthy control subjects (5 female; 30.9 ± 14 years, mean ± SD) free of musculoskeletal or neurological disorder were enrolled in the study after written consent was provided. Two individuals with chronic SCI had a neurological deficit grade A on the American Spinal Injury Association Impairment Scale (AIS), 2 had AIS B, 1 had AIS C, and 5 had AIS D, while the level of SCI ranged from C4 to T11. Individuals with motor complete SCI were included in order to assess potential changes in presence of minimal descending inputs. Both SCI and healthy control subjects participated in a previous study [10], and are identified here with the same code.

**Table 1:** Characteristics and demographics of individuals with spinal cord injury (SCI).

ID	Gender	Age (yrs)	Post injury (yrs)	Level of injury	AIS	Cause of injury	Motor score		Number of sessions	List of medication
							LL	RL		
R01	M	51	3.5	C7	B	Ocean wave-related	0	0	17	Baclofen 20 mg 4xD; Sertraline 60 mg 1xD; Oxybutynin 5 mg 3xD
R03	F	24	2	C6	D	Fall from height	20	5	11	Nitrofurantoin 100 mg 1xD; Amitriptyline 50 mg 1xD; Dextroamphetamine-Amphetamine 20 mg 2xD
R04	M	51	2	T5	D	Calcification of ligaments	18	18	15	Metaxalone 800 mg 4xD; Oxycodone 10–325 mg 4xD; Baclofen 200 mg 1-2xD; Diltiazem ER 240 mg 1xD; Valacyclovir 500 mg 3xW; Oxymorphone 40 mg 2xD; Omeprazole 20 mg 1xD
R06	M	36	4	T2	A	MVC	0	0	17	None
R07	F	39	16	T12	C	MVC	17	5	23	Baclofen 2 mg 2xD
R08	M	27	9	C7	B	MVC	0	0	18	Oxybutynin 10–15 mg 1xD
R09	F	19	5	T1	D	SX	21	12	15	None
R10	M	47	28	T7	A	GSW	0	0	14	None
R11	M	38	6	T9	D	GSW	23	23	18	Gabapentin 800 mg 3xD; Zenflox 200 mg 2xD; Baclofen 10 mg 3xD; Oxycodone-Acetaminophen 10 mg 3xD
R12	M	31	12	C6	D	MVC	25	17	18	None

Level of SCI corresponds to neurological level of injury. The American Spinal Injury Association Impairment Scale (AIS) is indicated for each subject based on sensory and motor evaluation per AIS guidelines. Motor scores (out of 25 maximal points for each leg) are indicated based on the manual muscle test of key muscles and evaluated as 0 = no contraction, 1 = flicker or trace of contraction, 2 = active movement with gravity eliminated, 3 = active movement against gravity, 4 = active movement against gravity and resistance, 5 = normal muscle power. The number of transspinal stimulation sessions given during the intervention is indicated for each participant. Medication was taken at similar times of day. MVC = Motor vehicle crash; SX = Surgery; GSW = Gunshot wound; xD = Times daily; xW = Times weekly; LL = left leg; RL = right leg.

### Noninvasive transspinal stimulation

With subjects seated, the T10 spinous process was identified via palpation and in consolidation with anatomical landmarks (T1 spinal process, end of sternum, and end of rib cage). A single reusable self-adhered cathode electrode (10.2 × 5.1 cm^2^, Uni-Patch, Massachusetts, USA) was placed at T10 equally spaced along the vertebrae ending at L1-2. Two interconnected electrodes (anode, same type as the cathode) were placed on either side of the abdominal muscles or iliac crests depending on each subject’s reported level of comfort or if stimulation caused bladder discomfort [7,8,10,17,18]. Stimulation was delivered by a constant current stimulator (DS7A or DS7AH, Digitimer, Welwyn Garden City, UK) that was triggered by Spike 2 scripts (Cambridge Electronics Design Ltd., Cambridge, UK). The position of the cathodal stimulating electrode was based on the presence of soleus TEP depression in response to paired transspinal stimuli delivered at an interstimulus interval (ISI) of 60 ms. After the optimal location was identified, the electrode was affixed to the skin via Tegaderm transparent film (3M Healthcare, Minnesota, USA), and maintained under pressure via a custom-made pad. To ensure consistency of stimulation site across sessions, the skin area was covered by Tegaderm film.

For all subjects, transspinal stimulation was delivered daily, excluding weekends and holidays, in a supine position. Knee and hip joints were flexed at 30°, ankles were supported in a neutral position, and legs were maintained in midline via external support. Similar to stimulation parameters we have previously used [10,17], transspinal stimulation was delivered as a 1-ms single monophasic square pulse at 0.2 Hz based on the postulated interactions between the motor evoked potentials and TEPs [18], and between the soleus H reflex and TEPs [7,11] at the spinal cord. Individuals with SCI received a total of 16.6 ± 1 stimulation sessions for an average of 60 ± 2 min per session (Table 1). Healthy control subjects received 10 stimulation sessions (40 ± 0.1 min per session) except one subject whom received 12 sessions because post-stimulation assessments were arranged to be performed during a weekday and not after 2-days of absent stimulation (e.g. weekend). Stimulation intensity was delivered in multiples of soleus TEP resting threshold established at baseline, and corresponded to the intensity needed to evoke a soleus TEP of 100 mV peak-to-peak amplitude. The soleus TEP resting threshold was 96.9 ± 24 mA and 28.9 ± 5.7 mA (*p* = .023) for individuals with and without SCI, respectively. Because the maximal TEP amplitudes decrease after 10–15 min of continuous transspinal stimulation [10,17], suprathreshold transspinal stimulation (15-min for SCI group; 10-min for control group) was alternated with subthreshold (5-min for both groups) stimulation. Suprathreshold intensities were based on 1.2 times soleus TEP resting threshold with incremental increases in intensity as the sessions progressed of 0–112 mA depending on the constant current stimulator being used. In healthy control subjects, stimulation intensities at each session ranged from 0.7 ± 0.1 to 10.3 ± 2.9 (6.4 ± 1.7) of the right soleus TEP resting threshold established at baseline, increasing from an average of 105.1 ± 21.5 mA in the first five sessions to an average of 130.3 ± 24.3 mA in the last five sessions. Similarly, in SCI subjects, intensities ranged from 0.4 ± 0.1 to 4.3 ± 0.9 (2.2 ± 0.4) of the right soleus TEP resting threshold established at baseline. Transspinal stimulation was well tolerated by all participants, blood pressure remained unaltered, and no adverse events were encountered during or after each stimulation session.

### Neurophysiological assessments before and 1 or 2 days after transspinal stimulation

#### EMG recordings

Surface EMG was recorded by single bipolar differential electrodes (MA300-28, Motion Lab Systems Inc., Baton Rouge, LA) from the left and right soleus muscles. EMG signals were amplified, filtered (10–1000 Hz), sampled at 2000 Hz via a 1401 plus (Cambridge Electronics Design Ltd., Cambridge, UK) or an analog-to-digital acquisition system (National Instruments, Austin, Texas, USA) and stored for offline analysis.

#### Soleus H-reflex excitability and spinal inhibition

The soleus H reflex was evoked according to methods we have previously employed in individuals with and without SCI [19,20]. With subjects seated and both feet supported by a foot rest, a stainless steel plate of 4 cm^2^ in diameter (anode electrode) was secured proximal to the patella. The optimal stimulation site was established via a hand-held monopolar stainless steel head electrode used as a probe [20], and corresponded to the site that the M wave had a similar shape to that of the H reflex at both low and high stimulation intensities, and group Ia afferents were excited at low stimulation intensities before motor axons. When the optimal site was identified, the monopolar electrode was replaced by a pre-gelled disposable electrode (SureTrace, Conmed, New York, USA) that was maintained under constant pressure throughout the experiment with athletic foam pre-wrap. The stimulation site was reconfirmed for the permanent monopolar cathode electrode, based on the previously described criteria.

With subjects seated, the soleus maximal M wave (Mmax) was first evoked via a constant current stimulator (DS7A, Digitimer Ltd., UK). Then, the stimulation intensity that evoked a H reflex on the ascending part of the recruitment curve was determined. This intensity evoked a soleus H reflex that was 31 ± 1.9 and 38 ± 1.8% of the Mmax in healthy control and SCI subjects, respectively. At this intensity, 15 soleus H reflexes were recorded randomly at 0.1, 0.125, 0.2, 0.33, and 1.0 Hz to establish changes in homosynaptic depression. Similar procedures were followed to establish changes in postactivation depression, during which 20 paired tibial nerve stimuli were delivered randomly at the ISIs of 60, 100, 300, and 500 ms at a constant stimulation frequency of 0.2 Hz.

Furthermore, the posterior tibial nerve at the popliteal fossa was stimulated at 0.2 Hz and at least 120 responses were recorded at varying stimulation intensities to assemble a soleus M-wave and H-reflex recruitment curve. Tibial nerve stimulation was delivered via a customized stimulator that the intensity was software controlled and adjusted by a few mA through a LabVIEW custom-made script, and saved along with the triggering pulses and EMG recordings. This experimental approach ensures that H reflexes are continuously evoked at different intensities and not as consecutive H reflexes at the same intensity.

In all subject groups, the same procedures were adapted for soleus H reflexes recorded from the left and right legs, which were performed on different days before and after cessation of transspinal stimulation. Results from each neurophysiological assessment are presented for each leg and as averages from both legs.

### Clinical assessments before and one day after transspinal stimulation

In individuals with SCI, the Penn spasm frequency and Penn severity scales, ankle clonus, and the modified Ashworth scale [21,22] were evaluated before and after repeated cathodal transspinal stimulation. Ankle clonus for both legs was evaluated as 0: absent clonus, 1: fatigable, 1–20 beats per minute, 2: fatigable, 20–100 beats per minute and 3: infatigable, continuous repeated clonus.

### Data analysis and statistics

The soleus M waves and H reflexes recorded at varying stimulation intensities (input-output curve) were measured as peak-to-peak amplitude of the non-rectified waveform, and were normalized to the associated Mmax to counteract differences of muscle geometry across subjects. A Boltzmann sigmoid function (Eq 1) was then fitted separately to the normalized soleus M waves and/or H reflexes plotted against non-normalized stimulation intensities [23,24]. The estimated parameters in Eq 1 denote the Mmax or maximal H reflex (Hmax), the slope parameter of the function (m), the predicted intensity corresponding to 50% of the Hmax (S50-Hmax) or Mmax (S50-Mmax), and the H-reflex or M-wave amplitude at a given stimulus value (H_(s)_ or M_(s)_). From the Boltzmann sigmoid function fitted separately to soleus M waves and/or H reflexes plotted against the stimulation intensities, we estimated the S50-Hmax and/or the S50-Mmax. The predicted S50-Hmax and S50-Mmax were used to normalize the stimulation intensities, and group the responses across subjects. The S50-Hmax observed at baseline was used to normalize the stimulation intensities that soleus H reflexes were recorded at different intensities before and after transspinal stimulation.

$$\text{M}(\text{s})=\frac{\text{Mmax}}{\left(1+\text{exp}\left(\text{m}(\text{S}50-\text{s})\right)\right)},\;\text{H}(\text{s})=\frac{\text{Hmax}}{\left(1+\text{exp}\left(\text{m}(\text{S}50-\text{s})\right)\right)}$$ (1)

Averages of normalized M waves were calculated in steps of 0.05 (up to 1.0 times the S50-Mmax) and 0.1 (>1.0 times the S50-Mmax), while H reflexes were grouped in steps of 0.1. The above described offline analysis was done separately for each soleus H-reflex recruitment curve assembled before and after transspinal stimulation. The normalized M waves and H reflexes were grouped separately for AIS C-D, AIS A-B and healthy control subjects based on multiples of stimulation intensities and time of testing.

For homosynaptic depression, soleus H reflexes evoked at 0.125, 0.2, 0.33, and 1.0 Hz for each subject and sweep were measured as the area under the full-wave rectified waveform, and normalized to the mean amplitude of the homonymous H reflex evoked at 0.1 Hz. For postactivation depression, the H2 (H reflex evoked by the 2^nd^ pulse) was normalized to the H1 (H reflex evoked by the 1^st^ pulse) within each pair of tibial stimuli and the average was estimated. Soleus H reflexes were grouped together for AIS C-D, AIS A-B, and healthy control subjects based on stimulation frequency and/or ISI and time of testing.

For each neurophysiological assessment and participant group, a repeated measures analysis of variance (ANOVA) was conducted separately for recordings taken from the left and right legs, as well as from both legs, to establish significant differences before and after repeated transspinal stimulation. A repeated measures ANOVA was applied to normalized soleus H reflexes recorded at different stimulation frequencies, upon paired tibial nerve stimuli at different ISIs, and also for M waves and H reflexes grouped based on multiples of S50-Mmax or S50-Hmax. Significant changes on clinical assessments were established with a paired *t*-test. For all statistical tests, the effects were considered significant when *p* < .05. Results are presented as mean values along with standard error (SE) unless otherwise stated.

## Results

### Soleus H-reflex excitability before and after repeated transspinal stimulation

**Fig 1:**
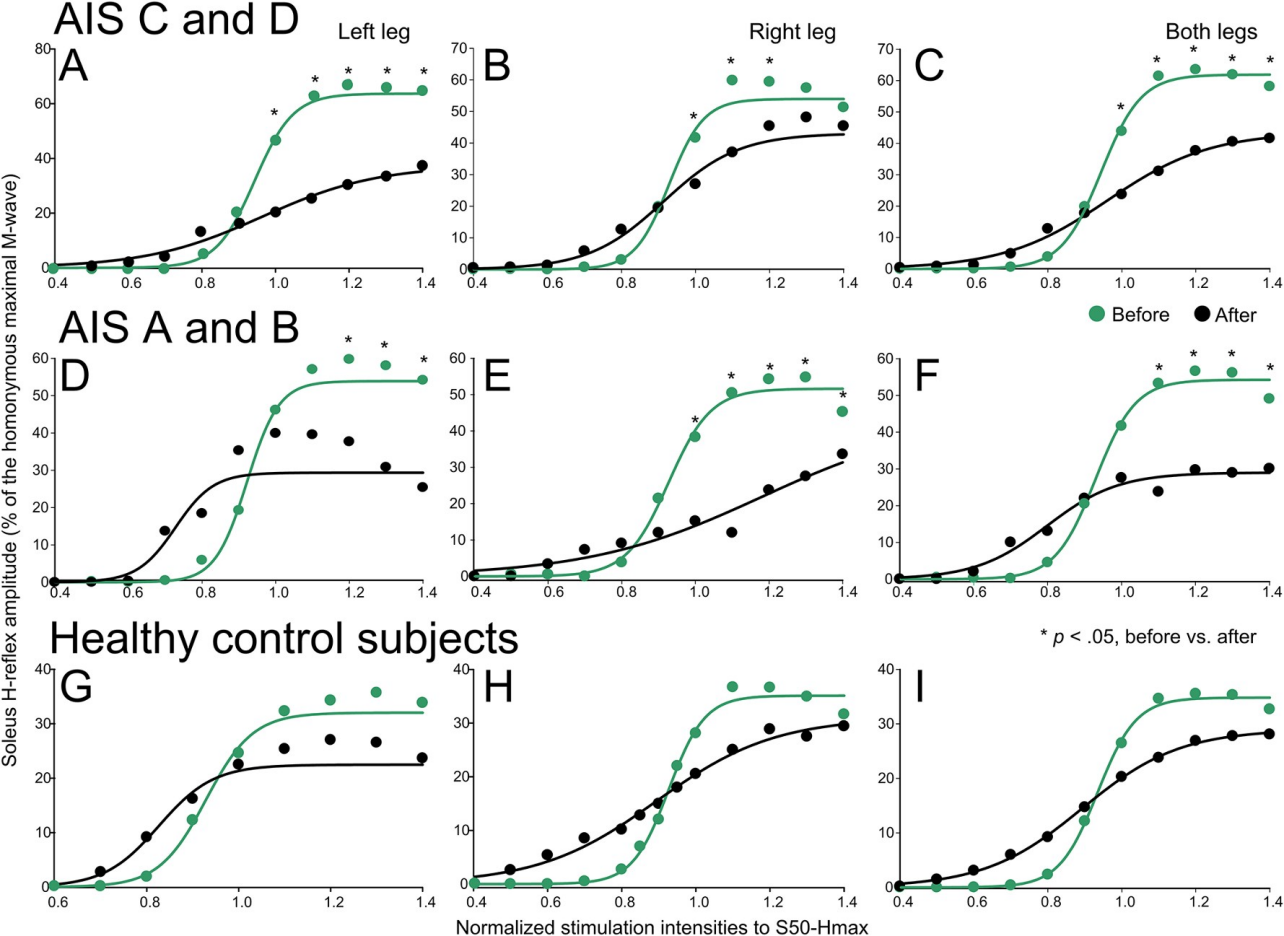
Soleus H-reflex recruitment curves before and after repeated transspinal stimulation in individuals with and without SCI. Soleus H reflexes recorded at increasing stimulation intensities until the H reflex reached maximal amplitudes as a percentage of the Mmax are plotted against the predicted 50% of maximal H-reflex stimulus intensity (S50-Hmax) observed at baseline. Soleus H reflexes before (green) and after (black) cathodal transspinal stimulation are indicated separately for the left and right legs, and from both legs for AIS C-D, AIS A-B, and healthy control subjects. *p < .05, significant differences of reflexes recorded before and after stimulation. Error bars not indicated for clarity purposes.

The soleus H-reflex recruitment curves before and after transspinal stimulation are shown against multiples of S50-Hmax and are indicated along with the sigmoid fit in Fig 1. Soleus H-reflex recruitment curves are indicated separately for the left and right legs, as well as from both legs, and are grouped for AIS C-D (Fig 1A–1C), AIS A-B (Fig 1D–1F), and healthy control (Fig 1G–1I) subjects.

In AIS C-D subjects, the left soleus H reflex (Fig 1A–1C) was significantly different before and after transspinal stimulation (F_1,117_ = 12.2, *p* < .001), while a significant interaction between time and intensities was found (F_11,117_ = 2.65, *p* = .005). Post hoc Bonferroni *t*-tests showed that the soleus H reflexes at intensities ranging from 1.0 to 1.4 multiples of S50-Hmax were significantly different before and after transspinal stimulation (for all *p* < .001). The right soleus H reflexes (Fig 1B) were significant different before and after transspinal stimulation (F_1,144_ = 5.26, *p* = .023), but an interaction between time and intensities was not found (F_11,144_ = 20.96, *p* = .22). Post hoc Bonferroni *t*-tests showed that the soleus H reflex at 1.0, 1.1 and 1.2 multiples of S50-Hmax was significant different before and after transspinal stimulation (for all *p* < .001). The soleus H reflexes, combined from both legs (Fig 1C), were significantly different before and after transspinal stimulation (F_1,288_ = 17.03, *p* < .001), while a significant interaction between time and multiples of S50-Hmax was found (F_11,288_ = 3.63, *p* < .001). Post hoc Bonferroni *t*-tests identified significant differences from 1.1 to 1.4 multiples of S50-Hmax (*p* < .001 for all).

In AIS A-B subjects, the left soleus H reflex (Fig 1D–1F) was significantly different before and after transspinal stimulation at 1.2, 1.3, and 1.4 multiples of S50-Hmax (F_1,40_ = 1.09, *p* = .30). Similarly, the right soleus H reflex (Fig 1E) was decreased after transspinal stimulation (F_1,68_ = 17.26, *p* < .001) while a significant interaction between time and intensities (F_11,68_ = 2.88, *p* = .004) was found. The soleus H reflexes, combined from both legs (Fig 1F), were significantly different before and after transspinal stimulation (F_1,136_ = 11.33, *p* < .001), while a significant interaction between time and stimulation intensities was found (F_11,136_ = 3.16, *p* < .001). Post hoc Bonferroni *t*-tests showed that the soleus H reflex at 1.1, 1.2 and 1.3 multiples of S50-Hmax was significantly different before and after transspinal stimulation (for all *p* < .001).

In healthy control subjects, the soleus H reflex (Fig 1G–1I) was not significant different before and after transspinal stimulation for the left (F_1,175_ = 0.183, *p* = .669) or right (F_1,278_ = 1.79, *p* = .18) legs. The soleus H reflexes, combined from both legs, remained unaltered after transspinal stimulation (Fig 1F; F_1,426_ = 2.04, *p* = .153). These results suggest that repeated transspinal stimulation decreased soleus H-reflex excitability in both motor incomplete and complete SCI, but remained unaltered in healthy control subjects whom have physiological reflex excitability.

**Fig 2:**
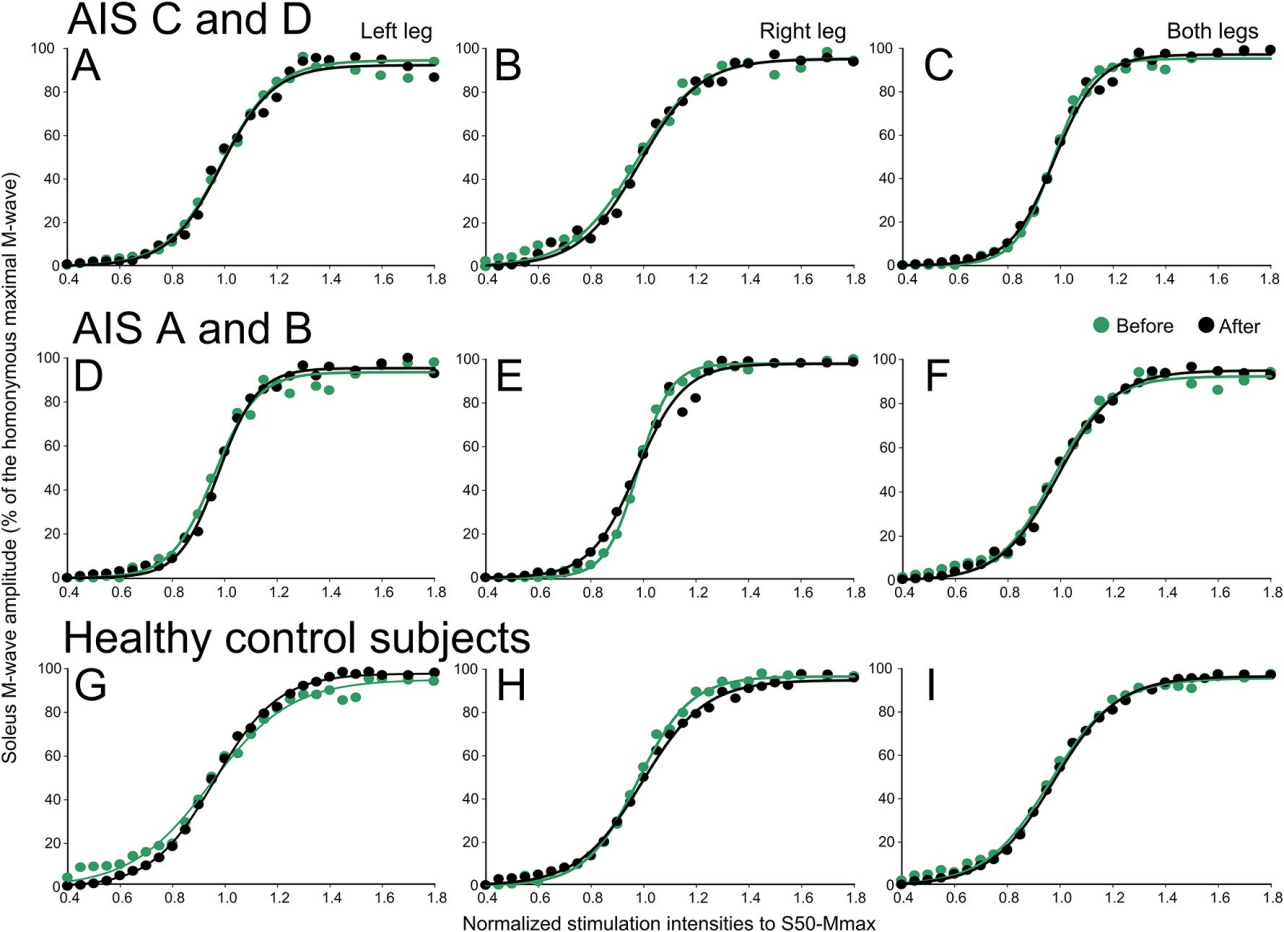
Soleus M-wave recruitment curves before and after repeated cathodal transspinal stimulation in individuals with and without SCI. Soleus M-wave sizes as a percentage of the Mmax are plotted against the predicted multiples of 50% of maximal M-wave stimulus intensity (S50-Mmax) for left and right legs grouped for AIS C- D, AIS A-B, and healthy control subjects. Error bars not indicated for clarity purposes.

The corresponding soleus M-wave recruitment curves before and after repeated transspinal stimulation along with the sigmoid fit are indicated in Fig 2. The soleus M-wave recruitment curves are indicated separately for the left and right legs, as well as from both legs, and are grouped for AIS C-D (Fig 2A–2C), AIS A-B (Fig 2D–2F), and healthy control (Fig 2G–2I) subjects. The M waves before and after transspinal stimulation for all cases were not significantly different (e.g. AIS A-D left leg: F_1,343_ = 0.01, *p* = .91; AIS A-D right leg: F_1,390_ = 1.57, *p* = .21), supporting that changes in soleus H-reflex excitability after transspinal stimulation could not be due to recruitment of different types of soleus motoneurons by Ia afferent volleys, and that stimulation and recording procedures were similar.

### Homosynaptic depression before and after repeated transspinal stimulation

**Fig 3:**
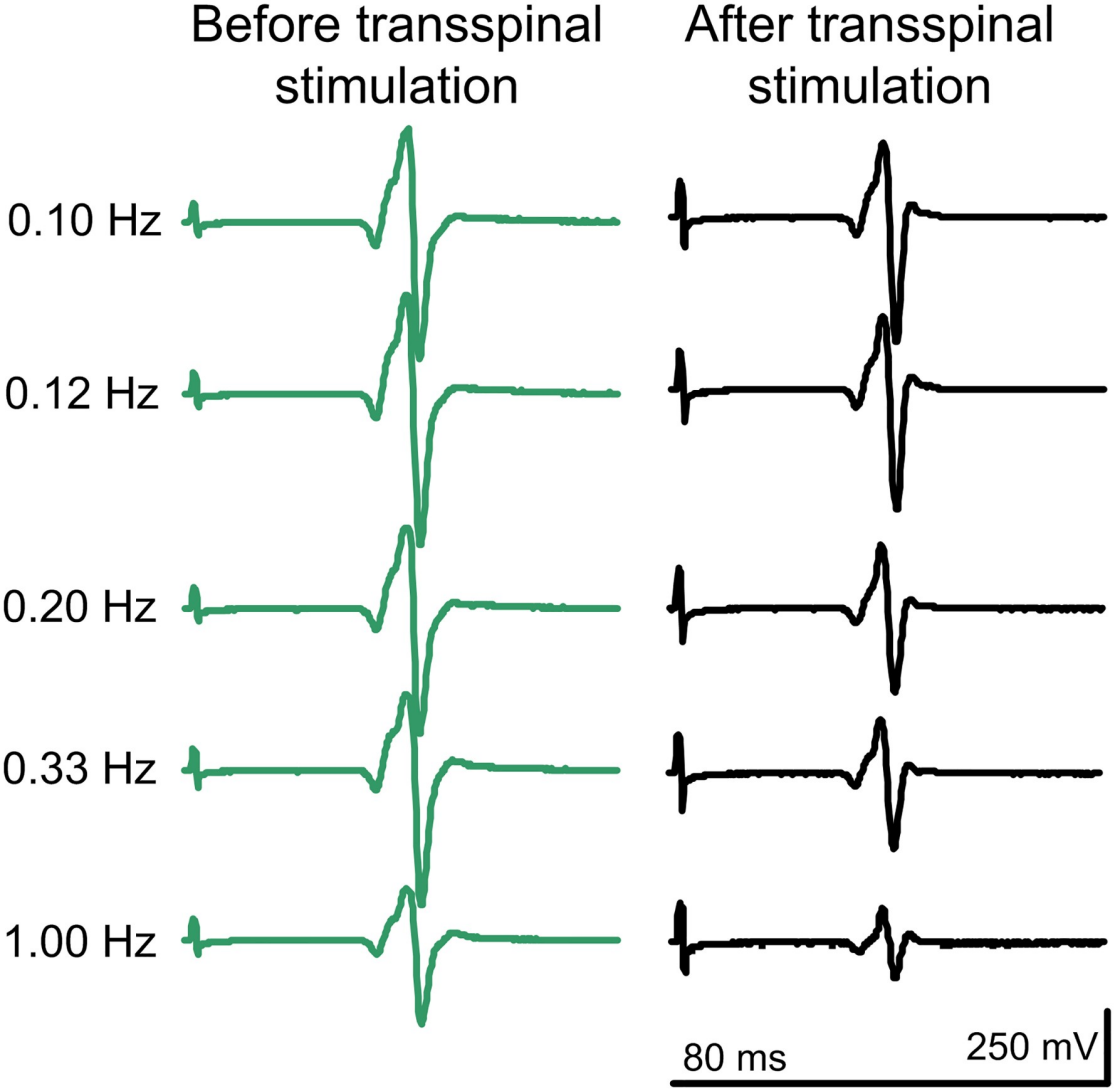
Soleus H-reflex waveforms at different stimulation frequencies in an individual with chronic SCI. Non-rectified waveform averages of soleus H reflexes recorded at different stimulation frequencies from an individual with SCI (R12—AIS D) in whom the soleus H reflex at 30% of the maximal M wave coincided with absent M wave.

Representative examples of non-rectified soleus H-reflex waveform averages recorded at different stimulation frequencies from one individual with SCI (R12—AIS D) before and after transspinal stimulation are indicated in Fig 3. It is evident that the soleus H reflex at 1.0 Hz is smaller compared to that evoked at 0.1 Hz, supporting for restoration of homosynaptic depression after transspinal stimulation in individuals with SCI.

**Fig 4:**
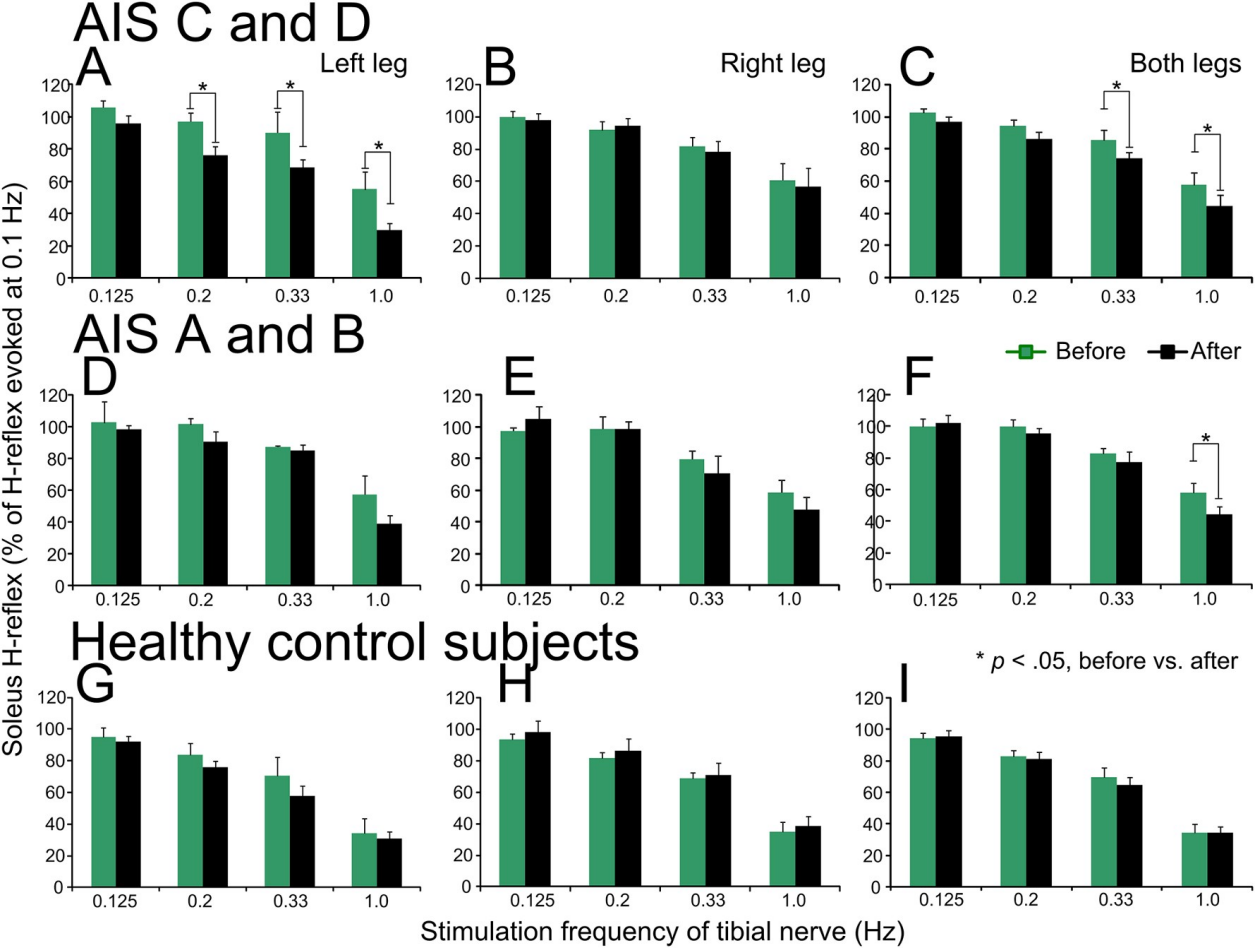
Soleus H-reflex homosynaptic depression before and after cathodal repeated transspinal stimulation in individuals with and without SCI. Soleus H reflexes evoked at different stimulation frequencies before (green) and after (black) repeated cathodal transspinal stimulation are indicated separately for the left, right and both legs combined for AIS C-D, AIS A-B, and healthy control subjects. On the abscissa, the stimulation frequency is indicated. Ordinate indicates the soleus H reflexes normalized to the H reflex evoked at 0.1 Hz. *p < .05, significant differences of reflexes recorded before and after stimulation. Error bars denote the SE.

The normalized soleus H-reflex amplitude recorded at different stimulation frequencies is indicated separately from the left and right legs, as well as average from both legs, before and after repeated transspinal stimulation in Fig 4. Soleus H reflexes are presented separately for AIS C-D (Fig 4A–4C), AIS A-B (Fig 4D–4F), and healthy control (Fig 4G–4I) subjects.

In AIS C-D subjects, the left soleus H reflex was significantly different before and after transspinal stimulation (F_1,40_ = 14.19, *p* < .001) and across stimulation frequencies (F_3,40_ = 23.42, *p* < .001). Post hoc Bonferroni *t*-tests showed that the H reflex was significant different at 0.2 Hz (t = 2.02, *p* = .04), 0.33 Hz (t = 2.08, *p* = .04), and 1.0 Hz (t = 2.45, *p* = .019) before and after transspinal stimulation (Fig 4A). In contrast, the right soleus H reflex was significantly different across stimulation frequencies (F_3,48_ = 12.89, *p* < .001) and not between the time of testing (F_1,48_ = 0.11, *p* = .73). The H reflexes, when combined from both legs, were significantly different before and after transspinal stimulation (Fig 4C; F_1,96_ = 7.28, *p* = .008) at stimulation frequencies of 0.33 and 1.0 Hz (F_3,96_ = 32.86, *p* < .001).

In AIS A-B subjects, the left soleus H reflex (Fig 4D) was not significantly different before and after transspinal stimulation (F_1,16_ = 3.00, *p* = .10) but differed significantly across stimulation frequencies (F_3,16_ = 21.59, *p* < .001). Similar results were also found for the right soleus H reflex (Fig 4E; time: F_1,24_ = 0.34, *p* = .56; frequencies: F_3,24_ = 19.9, *p* < .001). Post hoc Bonferroni *t*-tests showed that the H reflex evoked at 1.0 Hz was different from the H reflexes evoked at 0.125, 0.2, and 0.33 Hz (*p* < .05 for all). The H reflexes, combined from both legs, were significant different across stimulation frequencies (Fig 4F; F_3,48_ = 42.31, *p* < .001) and time of testing (F_1,48_ = 11, *p* = .04) with the H reflex at 1.0 Hz being significantly different before and after stimulation (t = 3.08, *p* = .04).

In healthy control subjects, the H reflex recorded at different stimulation frequencies (Fig 4G–4I) remained unaltered after transspinal stimulation in the left leg (F_1,72_ = 1.88, *p* = .17), right leg (F_1,72_ = 0.82, *p* = .36), and when H reflexes were combined from both legs (F_1,152_ = 0.2, *p* = .64). The soleus H-reflex amplitude varied significantly across stimulation frequencies (F_3,152_ = 64.85, *p* < .001).

Repeated measures ANOVA among subject groups, time of testing, and stimulation frequencies on the H reflexes combined from both legs showed that homosynaptic depression was not of similar strength across all three subject groups (F_2,296_ = 14.79, *p* < .001), being significantly different between AIS C-D and control (t = 4.45, *p* < .001), and between AIS A-B and control (t = 4.37, *p* < .001) subjects. Further, pairwise multiple comparisons showed that the soleus H reflex was significantly different between time of testing (F_1,296_ = 5.93, *p* = .015) for AIS C-D (t = 4.84, *p* < .001), AIS A-B (t = 2.25, *p* = .02), and healthy control (t = 2.24, *p* = .02) subjects. These results support that in chronic motor incomplete and complete SCI, the homosynaptic depression is present but of lesser strength compared to healthy control subjects, and that is potentiated after repeated transspinal stimulation in both motor incomplete and complete SCI.

### Postactivation depression before and after repeated transspinal stimulation

**Fig 5:**
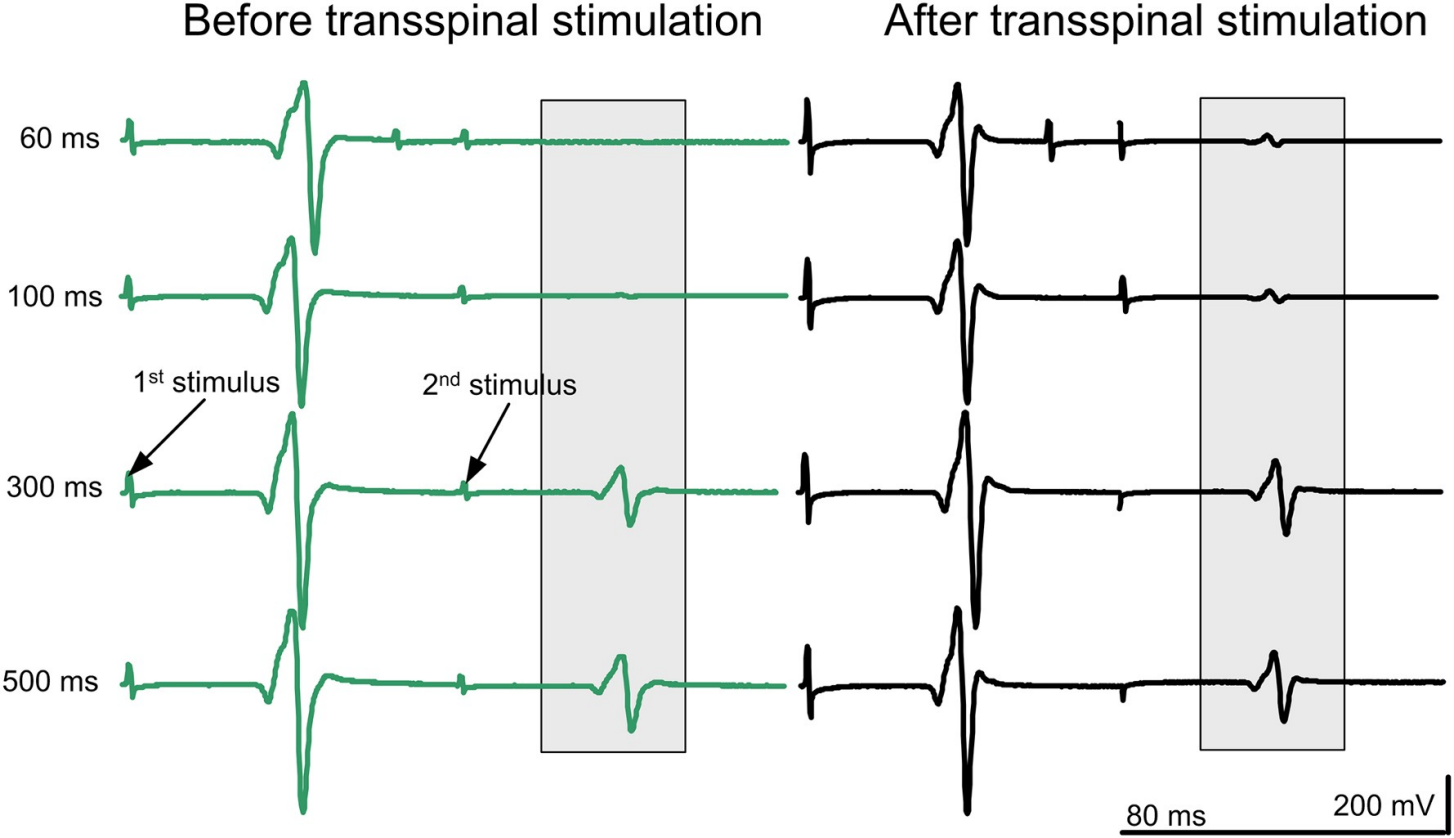
Soleus H-reflex waveforms following paired stimulation in an individual with chronic SCI. Non-rectified waveform averages of soleus H reflexes recorded upon paired tibial nerve stimuli from one individual with SCI (R12—AIS D) in whom the soleus H reflex at 30% of the maximal M wave coincided with absent M wave.

Representative examples of non-rectified soleus H-reflex waveform averages recorded upon paired tibial nerve stimuli at different ISIs from one individual with SCI (R12—AIS D) before and after transspinal stimulation are indicated in Fig 5. It is evident that the soleus H reflex was significantly depressed upon paired tibial nerve stimuli, supporting for presence of postactivation depression in people with SCI.

**Fig 6:**
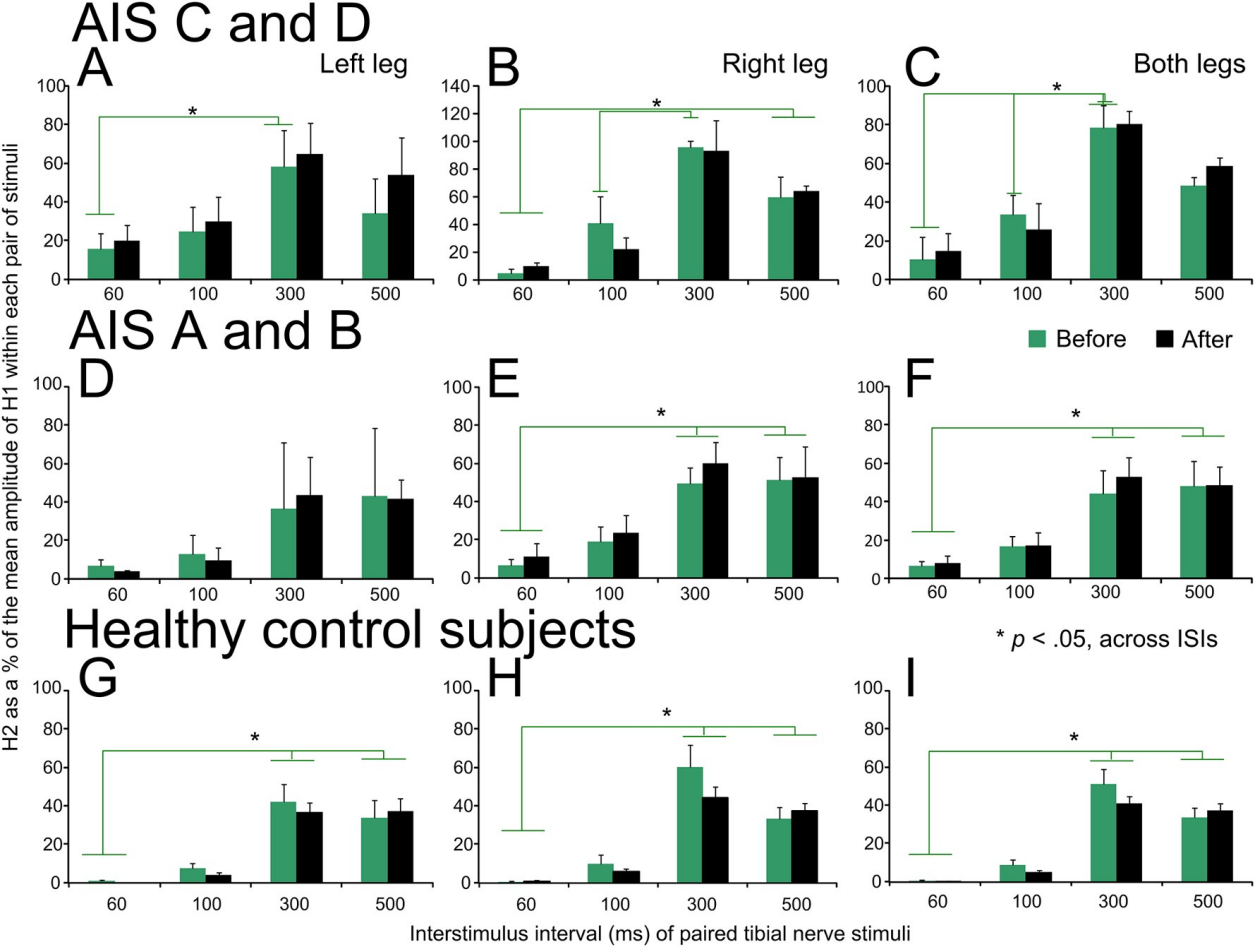
Soleus H-reflex postactivation depression before and after repeated cathodal transspinal stimulation in individuals with and without SCI. Soleus H reflexes evoked upon paired tibial nerve stimuli before (green) and after (black) repeated cathodal transspinal stimulation are indicated separately for the left and right legs, and from both legs for AIS C-D, AIS A-B, and healthy control subjects. On the abscissa, the interstimulus interval (ISI) of paired tibial nerve stimuli is indicated. Ordinate indicates the soleus H reflexes evoked by the 2^nd^ stimulus (H2) normalized to the mean amplitude of the H reflex evoked by the 1^st^ stimulus (H1). *p < .05, significant differences of reflexes recorded across different ISIs. Error bars denote the SE.

The normalized soleus H-reflex amplitude recorded separately from the left and right legs and as an average from both legs upon paired tibial nerve stimuli before and after repeated transspinal stimulation is indicated in Fig 6. Soleus H reflexes are presented separately for AIS C-D (Fig 6A–6C), AIS A-B (Fig 6D–6F), and healthy control (Fig 6G–6I) subjects.

In AIS C-D subjects, the left soleus H reflex was not significantly different before and after transspinal stimulation (F_1,35_ = 0.73, *p* = .39), but was significantly different across ISIs (F_3,35_ = 4.31, *p* = .01). Post hoc Bonferroni *t*-tests showed that the H reflex was significant different at the ISIs of 60 and 300 ms (t = 3.34, *p* = .002; Fig 6A). Similar results were also found for the right soleus H reflex (time: F_1,40_ = 0.09, *p* = .75; ISIs: F_3,40_ = 17.44, *p* < .001), while post hoc Bonferroni *t*-tests showed that the H reflex was significantly different among all ISIs (*p* < .05 for all) except between 60 and 100 ms (Fig 6B). When soleus H reflexes were combined from both legs (Fig 6C), similar results were found (time: F_1,128_ = 1.18, *p* = .27; ISIs: F_3,128_ = 87.63, *p* < .001).

In AIS A-B subjects, the left soleus H reflex (Fig 6D) was not significantly different before and after transspinal stimulation (F_1,13_ = 0.07, *p* = .99) or across ISIs (F_3,13_ = 2.94, *p* = .07). In contrast, the right soleus H reflex (Fig 6E) varied across ISIs (F_3,21_ = 11.61, *p* < .001) but was not significantly different before and after transspinal stimulation (F_1,21_ = 0.63, *p* = .43). Post hoc Bonferroni *t*-tests showed that the H reflex was significantly different among all ISIs (*p* < .05 for all) except between 60 and 100 ms and between 300 and 500 ms (Fig 6E). Similar results were found when soleus H reflexes were combined from both legs (Fig 6F; time: F_1,42_ = 0.26, *p* = .60; ISIs: F_3,42_ = 14.3, *p* < .001).

In healthy control subjects, the soleus H-reflex amplitude remained unaltered after transspinal stimulation in the left leg (F_1,60_ = 0.19, *p* = .66), right leg (F_1,60_ = 1.25, *p* = .26), and when H reflexes were combined from both legs (F_1,128_ = 1.18, *p* = .27), while the amount of depression varied across ISIs (*p* < .05).

Repeated measures ANOVA among subject groups, time of testing, and ISI on the H reflexes combined from both legs showed that postactivation depression was not of similar strength across all three subject groups (F_2,252_ = 24.3, *p* < .001), being significantly different between AIS C-D and AIS A-B groups (t = 3.39, *p* = .02), and between AIS C-D and control groups (t = 6.97, *p* < .001). These results support that in chronic motor incomplete and complete SCI, postactivation depression is present but of lesser strength compared to that observed in healthy control subjects, varies across ISIs, and remains unchanged after repeated transspinal stimulation.

### Clinically evaluated hyperreflexia before and after transspinal stimulation in individuals with SCI

In Table 2, the results of clinical assessments of hyperreflexia performed before and one day after cessation of repeated cathodal transspinal stimulation are indicated. The Penn spasm frequency, spasm severity and ankle clonus in both legs were significantly reduced after transspinal stimulation (*p* < .05 for all). Individuals with SCI reported improvements in urine control (R06), temperature regulation (R10), return of sweat (R06, R08), and less stiff leg muscles (R07, R08, R09).

**Table 2:** Clinical tests before and after repeated transspinal stimulation in SCI.

	Penn Spasm Frequency		Penn Spasm Severity		Ankle Clonus (Left leg)		Ankle Clonus (Right leg)	
Subject code	Before	After	Before	After	Before	After	Before	After
R01	1.00	2.00	1.00	2.00	3.00	3.00	3.00	3.00
R03	2.00	1.00	2.00	2.00	0.00	0.00	0.00	0.00
R04	2.00	2.00	3.00	3.00	1.00	1.00	1.00	2.00
R06	3.00	1.00	3.00	1.00	0.00	0.00	3.00	0.00
R07	3.00	2.00	3.00	1.00	3.00	0.00	3.00	0.00
R08	3.00	2.00	3.00	2.00	3.00	0.00	3.00	0.00
R09	2.00	1.00	3.00	2.00	0.00	0.00	0.00	0.00
R10	2.00	2.00	1.00	1.00	0.00	0.00	1.00	0.00
R11	3.00	1.00	2.00	2.00	3.00	0.00	3.00	1.00
R12	2.00	1.00	2.00	1.00	1.00	0.00	0.00	0.00
*p*-value	.004		.04		.04		.03	

For each subject, the self-reported Penn Spasm Frequency and Penn Spasm Severity are indicated before and after repeated cathodal transspinal stimulation. Ankle clonus for both left and right legs are indicated for before and after transspinal stimulation and evaluated as 0: absent clonus, 1: fatigable, 1–20 beats per minute, 2: fatigable, 20–100 beats per minute, 3: infatigable, continuous sustained clonus. *P*-values are indicated for before and after transspinal stimulation comparisons.

## Discussion

In this study, we probed spinal neuroplasticity after repeated cathodal transspinal stimulation via the soleus H reflex, a well-established neurophysiological biomarker of hyperreflexia and spasticity [20,25]. The major findings of this study are that transspinal stimulation 1) reduced soleus H-reflex excitability in both motor incomplete and complete SCI but not in healthy control subjects, 2) restored the amplitude of monosynaptic motoneuron responses following repetitive excitation of muscle spindle group Ia afferents at low stimulation frequencies (e.g. homosynaptic depression) regardless the severity of SCI, 3) did not affect postactivation depression in any of the subject groups, and 4) decreased the severity and frequency of spasms and ankle clonus. Consequently, transspinal stimulation alters spinally-mediated reflex actions and adjusts the responsiveness of motoneurons to proprioceptive afferent inputs in the injured human spinal cord.

Repeated transspinal stimulation resulted in significant depression of soleus H-reflex excitability in the left and right legs, and when reflexes were combined from both legs in motor incomplete and complete SCI, but remained unaltered in healthy control subjects whom have physiological reflex responses (Fig 1). The reduced soleus H-reflex excitability coincided with a leftward shift of the recruitment curve (Fig 1D) and stable M-wave recruitment curves (Fig 2). Further, the homosynaptic depression, which was present at baseline but of lesser strength in SCI subjects compared to healthy control subjects, was potentiated after repeated transspinal stimulation in both motor incomplete and complete SCI (Fig 4C and 4F). These neurophysiological changes were clinically manifested as decreased ankle clonus of both legs, and decreased self-reported changes in the severity and frequency of leg spasms (Table 2). These spinal reflex excitability changes can potentially be attributed to synaptic and non-synaptic neuroplasticity.

Homosynaptic depression is manifested as depressed motoneuron Ia excitatory postsynaptic potentials, and is attributed to decreased amounts of neurotransmitters released by the previously activated group Ia afferents at the Ia/motoneuron synapse [26–29]. In this study, we found that in SCI subjects homosynaptic depression was present but of lesser strength compared to that observed in healthy control subjects, and it was increased after repeated transspinal stimulation. Thus, we can suggest that transspinal stimulation restored homosynaptic depression in SCI subjects by affecting the amount of neurotransmitter being released by the stimulated group Ia afferents. Homosynaptic depression requires consequent activation of the same group Ia afferents, and occurs at the same Ia/motoneuron synapses with absent contribution from motor axons [30]. This type of inhibition, although not related to the classical presynaptic inhibition that involves activity of spinal inhibitory interneuron networks and parallel primary afferent depolarization [31], can be viewed as a spinal mechanism that gates proprioceptive inputs in resting individuals, and is regularly used as an indicator of calcium release probability. Among other mechanisms [32], malfunction of this gating mechanism in resting individuals can result in long periods of monosynaptic depolarization of motoneurons and thereby to hyperreflexia and spasms [19,25,33–35]. Spasticity and spasms after SCI have been partly associated with enhanced activation of calcium-mediated persistent inward currents [36–38], that under physiological conditions account for the systematic variation in input-output properties of motoneurons within a pool [39]. Therefore, repeated transspinal stimulation at intensities that produces depolarization of motoneurons over multiple segments can potentially have affected persistent inward currents exerted at the somata and dendrites of motoneurons. Possible neuroplasticity mechanisms also include potentiation of group Ia afferents hyperpolarization and changes in the concentration of ion channels at nerve terminals [40,41]. This is supported by the summation of the soleus H reflex and soleus TEP in the surface EMG when both responses are matched to depolarize the same soleus motoneurons, and neural interactions are confined to occur at the peripheral nerve axons based on the timing between the two stimuli [7,11].

However, the decreased reflex excitability and increased homosynaptic depression cannot be attributed exclusively to presynaptic mechanisms of neuroplasticity because the soleus H reflex at maximal amplitudes is not purely monosynaptic [20]. Further, postsynaptic receptor desensitization has been reported when successive stimuli are delivered to a presynaptic axon [42,43]. Consequently, we can propose that neurophysiological changes resulted by promotion of postsynaptic inhibition or depression of postsynaptic excitation. However, further research is needed to identify the specific group of interneurons and neuroplasticity site.

Soleus H-reflex postactivation depression, induced by paired tibial nerve stimuli, was present at baseline in both motor incomplete and complete SCI subjects, was of lesser strength compared to healthy control subjects, and remained unaltered after repeated transspinal stimulation in all three subject groups (Fig 6). At the ISI of 300 ms, the soleus H reflex was larger in amplitude compared to that observed in the remaining ISIs. Removal or reduced H reflex depression at 300 ms can be attributed to postactivation potentiation, changes in the neuromuscular junction due to antidromic propagating nerve impulses following repetitive activity, and decreased excitatory potentials at the neuromuscular junction [44,45]. Intercurrent facilitation by paired tibial nerve stimuli has been previously reported [46]. Specifically, a conditioning H1 stimulus on a testing H2 stimulus results in depression of H2 responses that is maximal at 225 ms, while recovery of reflex depression starts at 300 ms [46]. Because of the long latency, it was proposed that intercurrent facilitation of soleus motoneurons involves long loop reflex circuits [47,48]. Consequently, the soleus H-reflex postactivation depression resulting from paired stimuli at specific ISIs is likely mediated by different mechanisms compared to the soleus H-reflex homosynaptic depression. Regardless the neuronal mechanism, repeated transspinal stimulation did not affect soleus H-reflex postactivation depression in any of the subject groups (Fig 6), suggesting that this neurophysiological mechanism may not be suitable to detect neurorecovery after a therapeutic intervention in SCI.

Lastly, the results obtained in this study along with the parallel increases in the motor output of major extensor muscles in the same patients [10], support for similar neuroplasticity mechanisms to that reported after conventional activity-based therapies [24,49,50]. For example, locomotor training in both human and animal models decreases hyperreflexia by restoring spinal inhibition exerted on muscle spindle primary endings, and motoneuron excitability through recovery of neurotrophic factors and ion homeostasis [51–53]. Similar mechanisms have also been shown for soleus H-reflex operant conditioning protocols. Successful up and down-conditioning in rats and humans with SCI, respectively, improves locomotion and interlimb coordination by raising motoneuron firing threshold and increasing GABAergic terminals on motoneurons [54–56]. These studies suggest that activity-based interventions incorporating repeated motor activity or reflex normalization use similar pathways to that of transspinal stimulation. Consequently, transspinal stimulation can be used to complement conventional activity-based interventions in optimizing meaningful and long-lasting recovery from neurologic injury and disease.

### Limitations

Neurophysiological tests were not performed at different time points following cessation of stimulation. Thus, we cannot comment on the sustainability of neuroplasticity beyond 1–2 days. Further, we delivered transspinal stimulation with a single, 1-ms square pulse at 0.2 Hz based on our previous studies on the powerful transspinal conditioning effects on cortical and spinal neuronal circuits in healthy control and individuals with SCI [7,8,10,11,17,18]. Future studies are warranted to assess the time course of neuroplasticity while utilizing different stimulation frequencies.

### Conclusions

The results of this study provide evidence that repeated transspinal stimulation decreases soleus H-reflex excitability, restores homosynaptic depression, and reduces clinically assessed hyperreflexia in individuals with motor incomplete and complete SCI. The neurophysiological and clinical changes observed in this study coincided with increased motoneuron output of multiple spinal segments in the same patients [10]. From a functional perspective, the decreased hyperreflexia, decreased spasticity and increased motoneuron output may benefit neural motor control and improve functional tasks such as standing and walking.

## Supporting information

S1 Data Contains all data reported in this paper. (ZIP)
